# Mitochondrial Alterations in Peripheral Mononuclear Blood Cells from Alzheimer's Disease and Mild Cognitive Impairment Patients

**DOI:** 10.1155/2016/5923938

**Published:** 2016-01-06

**Authors:** A. Delbarba, G. Abate, C. Prandelli, M. Marziano, L. Buizza, N. Arce Varas, A. Novelli, F. Cuetos, C. Martinez, C. Lanni, M. Memo, D. Uberti

**Affiliations:** ^1^Diadem Ltd., Spin Off of Brescia University, Brescia, Italy; ^2^Department of Molecular and Translational Medicine, University of Brescia, Brescia, Italy; ^3^Department of Psychology, University of Oviedo, Plaza Feijoo s/n, 33003 Oviedo, Spain; ^4^Institute of Biotechnology, University of Oviedo, 33006 Oviedo, Spain; ^5^Cabueñes General Hospital, Calle Los Prados 395, Gijón, 33203 Asturias, Spain; ^6^Department of Experimental and Applied Pharmacology, Centre of Excellence in Applied Biology, University of Pavia, Viale Taramelli 6, 27100 Pavia, Italy

## Abstract

It is well recognized that mitochondrial dysfunction contributes to neurodegeneration occurring in Alzheimer's disease (AD). However, evidences of mitochondrial defects in AD peripheral cells are still inconclusive. Here, some mitochondrial-encoded and nuclear-encoded proteins, involved in maintaining the correct mitochondria machine, were investigated in terms of protein expression and enzymatic activity in peripheral blood mononuclear cells (PBMCs) isolated from AD and Mild Cognitive Impairment (MCI) patients and healthy subjects. In addition mitochondrial DNA copy number was measured by real time PCR. We found some differences and some similarities between AD and MCI patients when compared with healthy subjects. For example, cytochrome C and cytochrome B were decreased in AD, while MCI showed only a statistical reduction of cytochrome C. On the other hand, both AD and MCI blood cells exhibited highly nitrated MnSOD, index of a prooxidant environment inside the mitochondria. TFAM, a regulator of mitochondrial genome replication and transcription, was decreased in both AD and MCI patients' blood cells. Moreover also the mitochondrial DNA amount was reduced in PBMCs from both patient groups. In conclusion these data confirmed peripheral mitochondria impairment in AD and demonstrated that TFAM and mtDNA amount reduction could be two features of early events occurring in AD pathogenesis.

## 1. Introduction

Alzheimer's disease (AD) is the most common form of dementia among the elderly, characterized by progressive memory loss and cognitive decline. AD affects millions of people worldwide and the number of AD cases is going to increase with longer life expectancy. For almost twenty years, the beta amyloid cascade theory has dominated thinking and research efforts in the comprehension and cure of this disease [1]. This theory derived largely from the characterization of rare disease-causing mutations in three genes, which code for amyloid-*β* protein precursor (A*β*PP), Presenilin 1, and Presenilin 2, all linked to amyloid-*β* metabolisms [2]. By contrast with familial cases, sporadic forms of the disease are very common and represent nearly 95% of cases [3]. Although the amyloid cascade hypothesis has also been extrapolated to explain sporadic AD, it does not completely explain the excessive A*β*_42_ production in these patients. Recent findings suggest that pathological changes that occur in AD brain, such as synapses and neuronal loss, even excess beta amyloid production, could be causally induced by mitochondrial dysfunction and increased oxidative stress [4–7]. Different studies have demonstrated that mitochondrial levels and complex IV activity are affected by amyloid-*β* [5, 8, 9], while other findings show that mitochondrial dysfunction via reactive oxygen species (ROS) production enhanced A*β* levels in the central nervous system (CNS) [10].

On the other hand, peripheral tissues of AD patients, such as platelets—where no elevated A*β* levels were found—showed mitochondrial dysfunction with enhanced ROS formation and increased oxidative stress. In particular, many evidences from several studies have demonstrated a decrease in cytochrome C oxidase activity (also known as complex IV) in platelets from AD patients [11–13]. In accordance with these findings, platelets from AD patients showed decreased ATP levels and increased ROS levels [12]. In addition mitochondrial membrane potential (MMP) was found to be reduced in ageing and in AD platelets in comparison with younger cells [14]. However, platelets are not suitable to evaluate the influence of nuclear-encoded proteins in maintaining mitochondria functionality in response to either physiological needs or pathological conditions. To our knowledge only a few contradictory studies have evaluated the mitochondrial functionality in lymphocytes derived from sporadic AD patients: two previous reports found no changes in the respiratory chain complexes activity in AD lymphocytes [15, 16], while more recently Feldhaus et al. [17] demonstrated an altered activity of respiratory complexes II and IV in AD lymphocytes compared with healthy subject blood cells.

Crucial for the maintenance of proper mitochondrial health and function is an intricate bigenomic program, involving both nuclear and mitochondrial genomes [18, 19]. For example, replication and transcription of mitochondrial genome are regulated by the action of a combination of proteins encoded by the nucleus, including (a) the peroxisome proliferator activator receptor gamma-coactivator 1*α* (PGC-1*α*) [20], (b) the master regulator of mitochondrial biogenesis, (c) the mitochondrial transcription factor A (TFAM), (d) RNA polymerase (POLRMT), (e) the transcription factors 1 and 2 (TFB1, TFB2), and (f) the nuclear respiratory factors 1 and 2 (NRF1, NRF2). Furthermore, the functionality of mitochondrial respiratory chain depends on both nuclear and mitochondria encoded proteins, as part of the electron transport chain (ETC), and of the Krebs cycle, involved in ATP synthesis [21].

Therefore, to further investigate mitochondrial alterations in AD pathology, we focused on studying particular mitochondrial-encoded and nuclear-encoded protein levels in peripheral blood mononuclear cells (PBMCs) isolated from AD and Mild Cognitive Impairment (MCI) patients.

## 2. Materials and Methods

### 2.1. Subjects

Patients affected by Alzheimer's disease (20) and Mild Cognitive Impairment (24) and (30) healthy age-matched controls were enrolled at the Neurology Unit of Cabueñes Hospital, Asturias (Spain). Subjects received a diagnosis of probable or possible AD according to NINCDS/ADRDA criteria (National Institute of Neurological and Communicative Disorders and Stroke/Alzheimer's Disease and Related Disorders Association), whereas MCI diagnosis followed the criteria of Petersen et al. when there was evidence of memory impairment, preservation of general cognitive and functional abilities, and absence of diagnosed dementia. Furthermore, depending on the patient's clinical profile, other tests of personalized assessment were performed. Recruited healthy controls met the following criteria: (1) no history of past or current psychiatric or neurologic disorders and (2) a score of higher than 26 in the Mini-Mental State Examination (MMSE). All patients included in this study underwent neuroimaging and neuropsychological assessment following the American Academy of Neurology (AAN) recommendations. Subjects with other neurological and psychiatric diseases as well as patients with a history of alcohol or drug abuse were excluded from the study. Besides this, subjects with acute comorbidities were also excluded. In addition, none of the subjects were taking antioxidant supplements. The ethical committee approved the protocol of the study, including the follow-up visits, and written consent was obtained from all subjects or, where appropriate, their caregivers. The demographic and clinical characteristics of the donors are shown in Table 1(a). PBMCs were obtained by Ficoll fractions of fresh blood [22] and were further used to obtain total protein extracts and DNA samples.

In addition DNA samples derived from 276 patients with sporadic AD, 70 patients with MCI, and 248 healthy age-matched controls (Table 1(b)) were obtained from the Institute “Fondazione Casimiro Mondino” and “Santa Margherita” in Pavia and from Sant'Orsola Hospital in Brescia, Northern Italy. The details of the enrollments were reported in Lanni et al. [23, 24].

### 2.2. Mitochondrial Enzyme Activity

Citrate synthase activity was measured spectrophotometrically at 412 nm at 25°C in whole cell extracts using a citrate synthase assay kit (Sigma-Aldrich, St. Louis, MO). Cell homogenates were added to buffer containing 10 mM 5,5-dithiobis-2-nitrobenzoic acid, 10 mM oxaloacetate, l M EDTA, 30 mM acetyl CoA, 5 mM triethanolamine hydrochloride, and 0.1 M Tris-HCl pH 8.1. Citrate synthase activity was expressed as *μ*mol of citrate produced/min/mL. Cytochrome C oxidase activity was measured spectrophotometrically at 550 nm at 25°C in whole cell extracts using a cytochrome C oxidase assay kit (Sigma-Aldrich, St. Louis, MO). Cell homogenates were added to buffer containing 10 mM Tris-HCl pH 7.0, 250 mM sucrose, 0.22 mM ferrocytochrome c, and 0.5 mM DTT. Cytochrome C oxidase activity was expressed as units of oxidized ferrocytochrome c/min/mL per minute at pH 7.0 at 25°C.

### 2.3. Western Blot and Immunoprecipitation

Protein samples (30 *μ*g each) were electrophoresed in 10% Acrylamide Gel and electroblotted onto nitrocellulose membranes (Sigma-Aldrich, St. Louis, MO). Membranes were blocked for 1 h in 5% bovine serum albumin in TBS-T (0.1 m Tris-HCl, pH 7.4, 0.15 m NaCl, and 0.1% Tween 20) and incubated overnight at 4°C with primary antibodies (described below). Protein extracts were processed for Western blot analysis. Primary antibodies were anti-MnSOD (1 : 200, Sigma Aldrich) and anti-tubulin (1 : 1000, Sigma-Aldrich). IRDye near-infrared dyes-conjugated secondary antibodies (LI-COR, Lincoln, Nebraska, USA) were used. The immunodetection was performed using a dual-mode Western imaging system Odyssey FC (LI-COR Lincoln, Nebraska, USA). Quantification was performed using Image Studio Software (LI-COR, Lincoln, Nebraska, USA) and the results were expressed as a ratio between MnSOD and tubulin fluorescent signal.

To analyze nitrated MnSOD, we immunoprecipitated 3NT-proteins with *μ*MACS Protein A/G MicroBeads (MACS Technology, Miltenyi). Specifically, 50 *μ*g PBMC protein extracts were incubated on ice for 30 minutes with 50 *μ*L of the *μ*MACS Protein A/G MicroBeads coated with 2 *μ*g of anti-3NT antibody (Sigma-Aldrich, St. Louis, MO, USA). After the incubation period, the magnetizable immune complex was passed over a separation column and placed in the magnetic field of a MACS Separator. At this stage magnetically labeled proteins were retained in the *μ* columns, while other proteins were efficiently washed away with 5 washes using RIPA buffer. Subsequently elution buffer was added and nitrated proteins were collected in a fresh tube. Cell lysate, washes, and elution were loaded onto 10% SDS-PAGE gels, followed by immunoblotting the nitrocellulose membranes with anti-MnSOD antibody as shown above.

### 2.4. ELISA Immunoassay

For ELISA 70 *μ*g of nondenaturated protein extracts was diluted in PBS 1x pH 7.4 and coated on the ELISA microplate overnight at 4°C. The next day plates were saturated with 100 *μ*L/well of blocking solution (PBS pH 7.4, 0.1% Tween 20, and 3% bovine serum albumin (BSA)) and incubated for 1 h at room temperature, followed by 2 h incubation at 37°C with anti-PGC-1*α* (0.5 *μ*g/mL), anti-TFAM (0.5 *μ*g/mL), anti-cytochrome B (0.5 *μ*g/mL), or anti-cytochrome C (0.5 *μ*g/mL) antibodies. After washing with PBST (PBS pH 7.4 and 0.5% Tween 20), 0.1 mg/mL anti-mouse secondary antibody conjugated with peroxidase was incubated in each well for 1 h at room temperature. Finally, 100 *μ*L of TMB (3,3,5,5-tetramethylbenzidine) substrate was added and the reaction was stopped with 100 *μ*L 2 M sulfuric acid. Optical density was measured using a microplate reader at a wavelength of 450 nm. Data were extrapolated by a standard curve created with serial dilution of the corresponding recombinant protein and then were expressed as the median ± SEM. The experiments were performed in triplicate.

### 2.5. Mitochondrial DNA Analysis

Total DNA was extracted from PBMCs with QIAamp DNA extraction kit (Qiagen, Hilden, Germany), and mtDNA was amplified using primers specific for the mitochondrial cytochrome B (CYT B) gene. Mitochondrial DNA copy number was normalized to nuclear DNA copy number by amplification of the acidic ribosomal phosphoprotein P0 (Arbp/36B4) nuclear gene. Primer sequences were designed using Beacon Designer 2.6 software (Premier Biosoft International, Palo Alto, CA, USA). The primers used were the following: for CYTB forwards 5′-GCCTGCCTGATCCTCCAAAT-3′ and reverse 5′-AAGGTAGCGGATGATTCAGCC-3′, 36B4 forwards 5′-AGGATATGGGATTCGGTCTCTTC-3′ and reverse 5′-TCATCCTGCTTAAGTGAACAAACT-3′.

### 2.6. Statistical Analysis

Results were given as median ± SEM or mean ± SEM values, according to the experiment. Statistical significance of differences was determined by mean values of both *t*-test and one-way ANOVA, followed by the Bonferroni test. Significance was accepted for *p* < 0.05. Statistical analyses were performed using Graph Pad Prism (Graph Pad Software Inc., San Diego, CA, USA) version 4.0.

## 3. Results

### 3.1. Biochemical Properties of Mitochondria in PBMCs Derived from AD and MCI Patients

**Figure 1:**
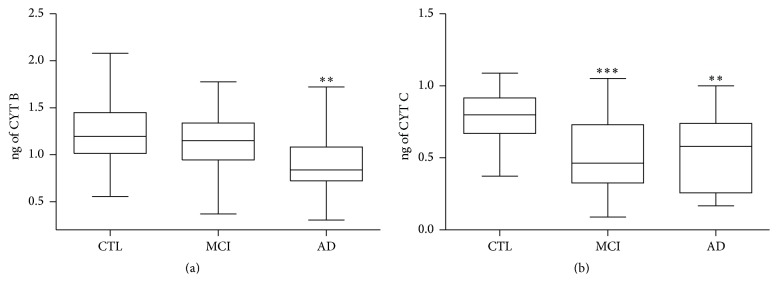
Mitochondrial protein levels in PBMCs derived from AD, MCI patients and healthy controls. Cytochrome B (CYT B) (a) and cytochrome C (CYT C) (b) levels were measured by ELISA in protein extracts derived from PBMCs. The values are expressed as ng of protein and are referred to as specific standard curves. Data was expressed as median ± SEM. The statistical significance was represented by the asterisks as follows: ^*∗∗*^ *p* < 0.001; ^*∗∗∗*^ *p* < 0.0001 versus corresponding control group.

PBMCs derived from 20 AD, 24 MCI patients and 30 age-matched controls were evaluated by measuring the protein levels of two redox cofactors, involved in the transfer of electrons through ETC complexes, and the activity of two enzymes, which gave the efficiency of mitochondrial functionality. In addition the amount of mitochondrial DNA (mtDNA) was also assessed. In particular, protein levels of cytochrome B (CYT B), a component of respiratory chain complex III [25], and cytochrome C (CYT C), a small heme-protein involved in the transfer of electrons between complexes III and IV [26], were evaluated in the three different groups by ELISA. As depicted in Figure 1(a), CYT B protein level was statistically reduced in AD, but not in MCI PBMCs (median ± SEM; CTL 1.19 ± 0.061 ng versus AD 0.84 ± 0.006 ng; *p* < 0.001). While CYT C was decreased in a significant manner in both AD and MCI, it is anyway lower in MCI when compared with healthy subjects (median ± SEM; CTL 0.89 ± 0.037 ng versus AD 0.58 ± 0.063 ng; *p* < 0.001; MCI 0.46 ± 0.060 ng; *p* < 0.0001).

**Figure 2:**
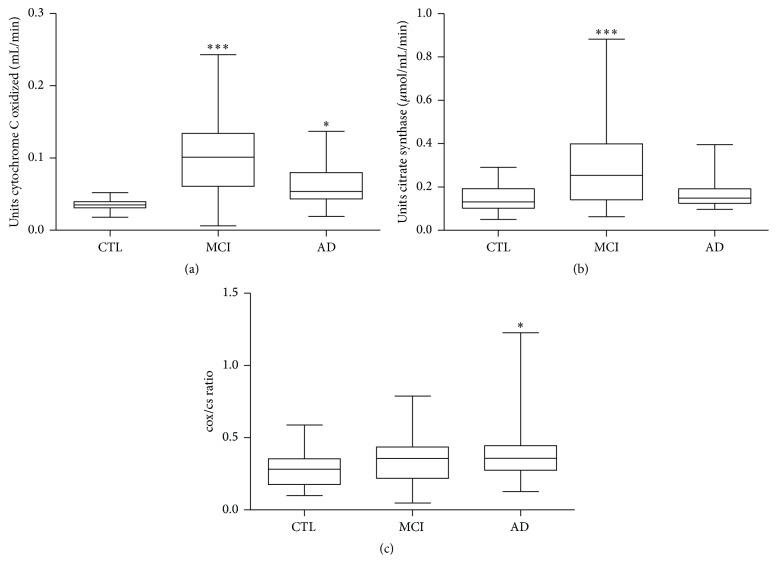
Mitochondrial protein activity in PBMCs derived from AD, MCI patients and healthy controls. Cytochrome C oxidase (a) and citrate synthase (b) activity was measured spectrophotometrically (see Section 2). The data were expressed as *μ*mole of enzyme produced/mL/min for citrate synthase activity and as units of enzyme oxidized/mL/min for cytochrome C oxidase. (c) Ratio between cytochrome C oxidase (cox) and citrate synthase (cs) was expressed as median ± SEM. The statistical significance was represented by the asterisks as follows: ^*∗*^ *p* < 0.05; ^*∗∗∗*^ *p* < 0.0001 versus corresponding control group.

Furthermore the mitochondrial functionality was estimated by measuring the activity of two enzymes, cytochrome C oxidase (complex IV) and citrate synthase, involved in oxidative metabolism and Krebs cycle, respectively. These two enzymes were found to be significantly higher in MCI subjects compared to the control group (median ± SEM; cytochrome C oxidase: CTL 0.035 ± 0.0015 units oxidized/mL/min versus MCI 0.10 ± 0.0013 units oxidized/mL/min; *p* < 0.0001; citrate synthase: CTL 0.131 ± 0.0011 *μ*mole produced/mL/min versus MCI 0.25 ± 0.033 *μ*mole produced/mL/min *p* < 0.0001) (Figures 2(a) and 2(b)). However, if cytochrome C oxidase activity was expressed as the ratio to citrate synthase, no differences were found between MCI and control groups (Figure 2(c)). At variance, AD group showed only a slight, but statistically significant increase of cytochrome C oxidase activity (median ± SEM; CTL 0.035 ± 0.0015 units oxidized/mL/min versus AD 0.15 ± 0.014 units oxidized/mL/min; *p* < 0.05), whereas no differences were observed in AD citrate synthase activity compared to controls (Figures 2(a) and 2(b)). As a result, also the cytochrome C oxidase/citrate synthase ratio was increased in these patients when compared with controls (median ± SEM; CTL 0.28 ± 0.02 versus AD 0.36 ± 0.06;  *p* < 0.05) (Figure 2(c)).

**Figure 3:**
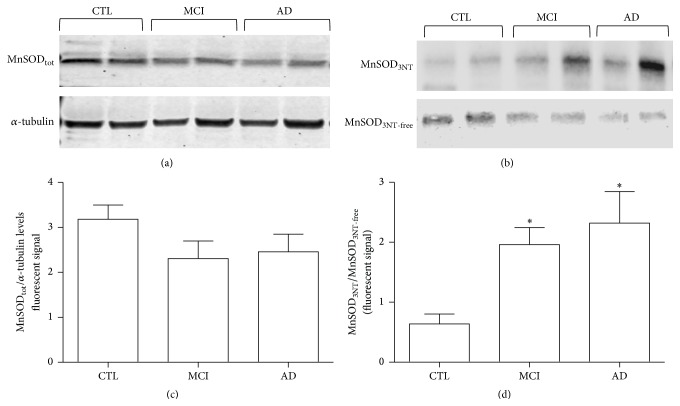
MnSOD level and its nitrated isoform in PBMCs derived from AD, MCI patients and healthy controls. Protein extracts derived from PBMCs of AD, MCI, and control were processed for Western blot analysis and immunoprecipitation assay (ip). (a) A representative immunoblot of protein extracts derived from 2 controls, 2 AD, and 2 MCI carried out with anti-MnSOD antibody. Tubulin was used to normalize the samples. (b) The 3NT-enriched fractions and 3NT-free washed fractions derived from ip experiments of the same 2 controls, 2 AD, and 2 MCI were loaded onto 12% SDS-PAGE gels and immunoblotted with anti-MnSOD antibody. (c) Quantitative analysis of MnSOD levels of 6 AD, 6 MCI, and 6 control samples expressed as MnSOD expression over *α*-tubulin levels. (d) Quantitative analysis of MnSOD nitrated isoform over 3NT-free isoform performed on 6 AD, 6 MCI, and 6 control samples. Data were expressed as mean ± SEM. The statistical significance was represented by the asterisk ^*∗*^ *p* < 0.05 versus the corresponding control group.

Since electron transport chain efficiency was directly correlated with the ROS generation as a byproduct of respiratory metabolism [27], and with the efficiency of antioxidant response, MnSOD enzyme was also evaluated. In particular MnSOD expression was studied by Western blot analysis (WB) using an anti-MnSOD monoclonal antibody on 6 CTL, 6 AD, and 6 MCI samples. In addition an immunoprecipitation experiment (ip) with an antibody that recognized the nitrated tyrosine residues (anti-3NT), followed by a WB with anti-MnSOD, was performed on the same samples. Figures 3(a)-3(b) show a representative WB and ip of 2 CTL, 2 AD, and 2 MCI samples. The amount of MnSOD (MnSOD_tot_) did not significantly differ among controls and AD and MCI patients, although its levels tended to be higher in controls than in AD and MCI patients (Figures 3(a)–3(c)). The expression of MnSOD_3NT_, evaluated as the ratio between the nitrated and 3NT-free isoform, was found to be higher in the MCI and AD in comparison with control samples (Figure 3(b)). The analysis of fluorescence signals of all samples examined (6 CTL, 6 AD, and 6 MCI) showed a significant enhancement of MnSOD_3NT_ in both AD and MCI patients when compared with controls (Figure 3(d)).

**Figure 4:**
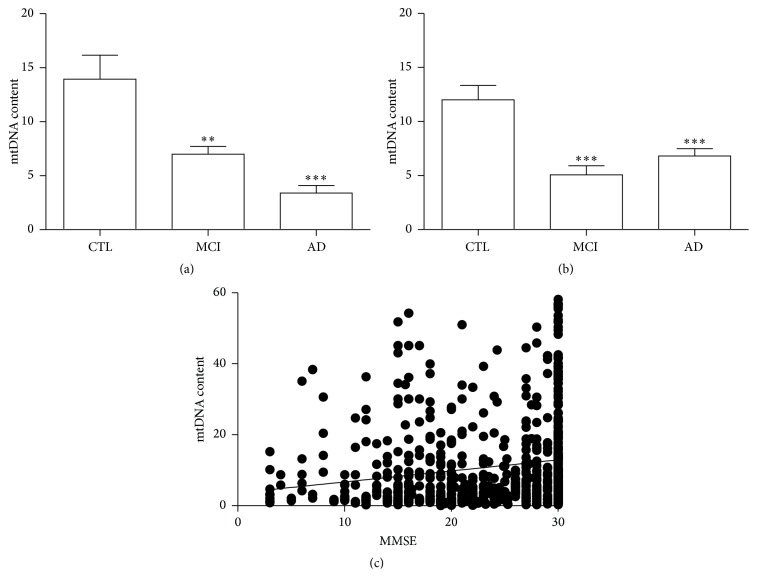
mtDNA copy content in PBMCs derived from AD, MCI patients and healthy controls. mtDNA content was measured as the amount of cytochrome B copy number (mitochondrial DNA) normalized to 36B4 gene copy number (nuclear DNA) by real time PCR in Spanish (a) and Italian (b) DNA samples. Data was expressed as mean ± SEM. The statistical significance was represented by the asterisks as follows: ^*∗∗*^ *p* < 0.001; ^*∗∗∗*^ *p* < 0.0001 versus the corresponding control group. The values of mtDNA content of both Spanish and Italian cohort were also correlated with the corresponding values of MMSE score (c). mtDNA content/MMSE (*p* = 0.0002 and *r* ^2^ = 0.03).

Another parameter evaluated to study mitochondria status was the mitochondrial DNA content, measured as the amount of mitochondrial DNA copy number normalized to nuclear DNA copy number [19]. In particular, the amplification of cytochrome B (mitochondrial gene) and 36B4 (nuclear gene) was obtained by real time PCR. The amount of mtDNA was found to be lower in AD and MCI patients in comparison with the control group (mean ± SEM; CTL 13.93 ± 2.26 versus AD 3.43 ± 0.69; *p* < 0.0001; CTL 13.93 ± 2.26 versus MCI 6.99 ± 0.71; *p* < 0.001) (Figure 4(a)).

mtDNA content was also measured in DNA samples derived from a larger cohort, composed of 248 healthy aged-matched controls and 70 MCI and 276 AD patients [23, 24]. As depicted in Figure 3(b), mtDNA content was significantly lower in AD and MCI patients than in the control group, confirming the results above (mean ± SEM; CTL 12.00 ± 3.73 versus AD 6.82 ± 2.85; *p* < 0.0001; CTL 12.00 ± 3.73 versus MCI 5.05 ± 2.39; *p* < 0.0001) (Figure 4(b)).

Finally, we attempted a correlation between mtDNA content and MMSE, considering both recruitments altogether. Loss of mtDNA content positively correlated with cognitive decline, measured as MMSE score (*r* ^2^ = 0.034,  *p* = 0.0002) (Figure 4(c)).

### 3.2. Expression of PGC-1*α* and TFAM in PBMCs Derived from AD and MCI Patients

**Figure 5:**
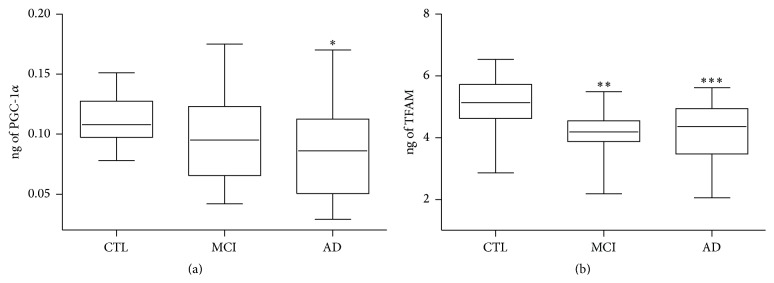
PGC-1*α* and TFAM protein levels in PBMCs derived from AD, MCI patients and healthy controls. PGC-1*α* (a) and TFAM (b) expression was measured by ELISA in protein extracts derived from PBMCs. The values are expressed as ng of protein and are referred to as specific standard curves. Data was expressed as median ± SEM. The statistical significance was represented by the asterisks as follows: ^*∗*^ *p* < 0.05; ^*∗∗*^ *p* < 0.001; ^*∗∗∗*^ *p* < 0.0001 versus the corresponding control group.

It is well established that the maintenance of mitochondria machine is under the control of nuclear transcription factors in a hierarchical structure [28, 29]. In order to investigate a possible role of nuclear-encoded proteins in mitochondrial impairment in AD and MCI blood cells, we evaluated the protein levels of PGC-1*α* and TFAM, by ELISA assay, in the three groups. Specifically, PGC-1*α* is a positive regulator of mitochondrial biogenesis and respiration that increases mitochondrial function and minimizes the build-up of byproducts, while TFAM is a target gene of PGC-1*α* that regulates replication and transcription of the mitochondrial genome and its expression is directly correlated with the mtDNA content [28, 29]. As shown in Figures 5(a) and 5(b) a statistically significant reduction of PGC-1*α* and TFAM was found in AD patients if compared with age-matched controls (median ± SEM PGC-1*α*: CTL 0.11 ± 0.02 ng versus AD 0.08 ± 0.04 ng;  *p* < 0.05; TFAM: CTL 5.16 ± 0.74 ng versus AD 4.12 ± 0.94 ng;  *p* < 0.0001). Interestingly MCI PBMCs showed a significant decrease of TFAM protein expression (median ± SEM, CTL 5.16 ± 0.74 ng versus MCI 3 ± 0.81 ng; *p* < 0.001), while PCG-1*α* was unchanged when compared with control samples (Figures 5(a) and 5(b)).

### 3.3. Cognitive Decline versus Mitochondrial Markers

**Figure 6:**
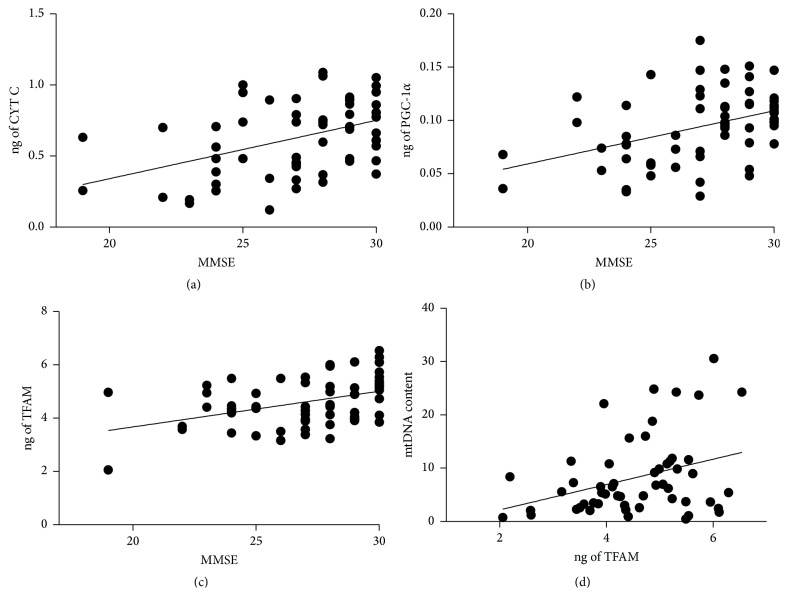
Correlation between CYT C, PGC-1*α*, or TFAM proteins with cognitive decline. The values of CYT C (a), PGC-1*α* (b), or TFAM (c) in protein extracts derived from PBMCs were correlated with the corresponding values of MMSE score. ng of CYT C/MMSE, *r* ^2^ = 0.189, *p* = 0.0006; ng of PGC-1*α*/MMSE, *r* ^2^ = 0.171, *p* = 0.0008; ng of TFAM/MMSE, *r* ^2^ = 0.175, *p* = 0.0009. (d) The values of TFAM were also correlated with the corresponding values of mtDNA content (*r* ^2^ = 0.117, *p* = 0.01).

In order to give more insight into the relationship between the progression of the disease and mitochondrial impairment, correlation studies between cognitive status and the markers reported above were performed. No association between CYT B and cognitive decline was found (data not shown). At variance, CYT C, PGC-1*α*, or TFAM expression positively correlated with MMSE score in a statistically significant manner (CYT C: *r* ^2^ = 0.189; *p* = 0.0006; PGC-1*α*: *r* ^2^ = 0.171; *p* = 0.0008; TFAM: *r* ^2^ = 0.175; *p* = 0.0009) (Figures 6(a)–6(c)). In addition, TFAM also correlated with mtDNA content (*r* ^2^ = 0.117; *p* = 0.01), confirming its role in regulating mtDNA transcription (Figure 6(d)).

## 4. Discussion

The arrangement of the mitochondria machine requires the well-coordinated action of both mitochondrial and nuclear proteins. In fact, the modulation of the electron transport respiratory chain, and accordingly of energy production, is the result of a crosstalk between mitochondria and the signals derived from the cellular environment. So, for example, cellular pathways involved in inflammatory and calcium signaling modulate mitochondrial activities and mitochondrial mass [30–38]. Furthermore ROS have been proposed as one of the signaling molecules that induce mitochondria activities via PCG-1*α* protein enhancement [39, 40].

Here we investigated whether nuclear and mitochondrial-encoded proteins, involved in both mitochondrial activity and the maintenance of mitochondrial DNA, were involved in mitochondrial dysfunction in AD pathology.

In particular we demonstrated that both CYT B and CYT C were compromised in PBMCs of AD patients, while only CYT C was found decreased in MCI blood cells if compared with age-matched controls, suggesting a progressive mitochondria impairment in the development of the disease. CYT B and CYT C, of mitochondrial and nuclear origin, respectively, are key cofactors of mitochondria machine participating in the transfer of electrons through complexes III and III-IV. Interestingly decreased CYT C expression positively correlated with the cognitive decline, measured as MMSE score.

Surprisingly, MCI PBMCs showed higher activities of two enzymes regulating the energy machine, cytochrome C oxidase and citrate synthase, involved in the respiratory chain processes and Krebs cycle, respectively. On the other hand the ratio of cytochrome C oxidase to citrate synthase, that better correlate the data with the mitochondria number, did not differ between MCI and control groups. Differently AD cells showed a slight increase of cytochrome C oxidase activity, confirmed also by the ratio of cytochrome C oxidase/citrate synthase. An increase of cytochrome C oxidase activity in different brain areas of AD mice overexpressing beta amyloid was also demonstrated by Strazielle et al. [41]. On the other hand, it must be stressed that contradictory results were reported regarding the activity of respiratory chain complexes in peripheral cells. For example, Feldhaus et al. [17] showed an increased energy metabolism in lymphocytes derived from AD, while Valla et al. [11] found a reduction in the activity of complexes III and IV in mitochondria isolated from blood platelets of AD patients. In our opinion, such discrepancies might be due to the different cellular phenotypes examined as well as to the different grades of illness severity.

Furthermore a significant statistical increase of the MnSOD nitrated isoform was observed in the AD and MCI groups, although the levels of the antioxidant enzyme in the two groups did not substantially differ from controls. A long lasting exposure to a prooxidant environment could be responsible for the MnSOD nitrated isoform enhancement. In line with our data, different authors reported that neurons derived from APP/PS1 tg mice showed decreased MnSOD activity, due to the nitration of its tyrosine residues [42, 43]. Mitochondria are the major sources of intracellular ROS, but they are also particularly vulnerable to oxidative stress [42]. We recently demonstrated, in immortalized lymphocytes derived from both familiar and sporadic AD, an increased nitrosative stress that affected protein function. In particular, nitration at tyrosine residues of the p53 protein compromised its wild type tertiary structure as well as its function [44]. Thus, although ROS act as signaling molecules to activate MnSOD via SIRT3, a PGC1-*α*-target, as a compensatory mechanism [39], it is also true that a sustained exposure to a prooxidant environment leads to a loss of adaptive response.

We also measured the amount of mtDNA in DNA samples from two different cohorts. The mtDNA content was found to be statistically decreased in MCI and AD of both groups if compared with control samples. However, it has to be stressed that the mean values of the Spanish MCI mtDNA content were found to be higher than that found in the Italian MCI DNA samples. This may be due to the different degree of cognitive decline in the two MCI groups; in fact the mean MMSE score was two points higher in the Spanish MCI compared with Italian patients. The number of mitochondria is cell specific and varies depending on the energetic requirement of the cells. It is also influenced by many factors, including the environmental and redox balance of the cell, the differentiation stage, and the number of cell signaling mechanisms [44–46]. Malik and Czajka [47] proposed the theory that the mtDNA content could be a biomarker of mitochondrial dysfunction. The premise of this theory is that values of mtDNA copy number, related to the value of nuclear DNA content, of a particular cell, normally within a healthy range, could change in condition of oxidative stress: the initial response to increased oxidative stress would be an adaptive response where the ratio between mtDNA and nuclear DNA would increase as a result of increased mitochondria biogenesis; persistent oxidative stress may lead to the depletion of mtDNA alongside mitochondrial dysfunction, resulting from damaged mtDNA and proteins. According to this theory, loss of mtDNA positively correlated with the cognitive decline, measured as MMSE score.

It is well established that the maintenance of mtDNA is under the control of nuclear transcription factors. The core machine of mitochondrial gene expression consists of TFAM, RNA polymerase *γ* (POLRMT), and mitochondrial transcript factor B2 (TFB2). TFAM has an additional role in packaging of mtDNA and it is necessary for mtDNA maintenance [48, 49]. TFAM is under the control of PGC-1*α*, which is upstream of all the pathways that regulate the expression of nuclear-encoded mitochondrial factors, and for this reason it is called the “master regulator” of mitochondrial biogenesis. In this study PGC-1*α* was found to be reduced only in AD but not in MCI PBMCs. In accordance with our findings, an involvement of PGC-1*α* in AD pathology was demonstrated in transgenic AD models and the ectopic expression of PGC-1*α* in a cell model of AD ameliorated their phenotype [50–52]. Nevertheless more importantly we found that TFAM expression was already significantly lower in MCI blood cells when compared with control PBMCs, suggesting its involvement in early stage of AD pathology. It is noteworthy that independent studies have demonstrated TFAM-gene variation as a moderate risk factor for AD development [53]. TFAM polymorphism Ser12Thr, that affects its function, was more highly common in the AD patients compared with healthy control groups [54].

Furthermore both PGC-1*α* and TFAM well correlated with cognitive decline. In addition TFAM positively correlated also with mtDNA content, confirming its important role in the regulation of mitochondrial genome.

Consistent evidence demonstrates that mitochondrial failure affecting replication and transcription of mtDNA is a feature of many ageing-related disorders: from cardiovascular to neurodegenerative diseases, suggesting that probably a common mechanism could be involved [55, 56]. The fact that such alterations were appreciated in the early stages of AD might help in the differential diagnosis supporting the current neuropsychological tests. However, further studies on a large number of patients have to be performed to better understand if TFAM reduction and decreased mtDNA content could be potentially a blood-based signature of AD.

## Figures and Tables

**Table 1a:** (a) Spanish cohort

	CTL	MCI	AD
*n* (M; F)	30 (16; 14)	24 (14; 10)	20 (8; 12)
Mean age ± SD (years)	71 ± 8	73 ± 6	74 ± 7
MMSE	29 ± 1	27 ± 2	24 ± 3
CDR	0	0.5	1
Disease onset		71.7 ± 6.49	71.67 ± 8.27
LOI (months)		24.5 ± 14.36	26.8 ± 13.25

**Table 1b:** (b) Italian cohort

	CTL	MCI	AD
*n* (M; F)	248 (116; 131)	70 (44; 26)	276 (99; 177)
Mean age ± SD (years)	75 ± 9.98	71 ± 8.71	78.23 ± 7.37
MMSE	29 ± 1	25.3 ± 4.45	15.1 ± 6.23
CDR	0	0.52 ± 0.06	2 ± 1
Disease onset		69.6 ± 6.2	72.5 ± 7.5
LOI (months)		30.2 ± 12.3	43 ± 31

AD: Alzheimer's disease; CTL: control; F: female; M: male; MMSE: Mini-Mental State Examination; CDR: Clinical Dementia Rating; LOI: length of illness; and *n*: number. Data are expressed as mean ± SD.

## References

[1] Hardy J. A., Higgins G. A. (1992). Alzheimer's disease: the amyloid cascade hypothesis. *Science*.

[2] Hardy J., Selkoe D. J. (2002). The amyloid hypothesis of Alzheimer's disease: progress and problems on the road to therapeutics. *Science*.

[3] Ray S., Britschgi M., Herbert C. (2007). Classification and prediction of clinical Alzheimer's diagnosis based on plasma signaling proteins. *Nature Medicine*.

[4] Pérez-Gracia E., Torrejón-Escribano B., Ferrer I. (2008). Dystrophic neurites of senile plaques in Alzheimer's disease are deficient in cytochrome c oxidase. *Acta Neuropathologica*.

[5] Rhein V., Song X., Wiesner A. (2009). Amyloid-*β* and tau synergistically impair the oxidative phosphorylation system in triple transgenic Alzheimer's disease mice. *Proceedings of the National Academy of Sciences of the United States of America*.

[6] Reddy P. H. (2009). Amyloid beta, mitochondrial structural and functional dynamics in Alzheimer's disease. *Experimental Neurology*.

[7] Reddy P. H., Beal M. F. (2008). Amyloid beta, mitochondrial dysfunction and synaptic damage: implications for cognitive decline in aging and Alzheimer's disease. *Trends in Molecular Medicine*.

[8] Hauptmann S., Scherping I., Dröse S. (2009). Mitochondrial dysfunction: an early event in Alzheimer pathology accumulates with age in AD transgenic mice. *Neurobiology of Aging*.

[9] David D. C., Hauptmann S., Scherping I. (2005). Proteomic and functional analyses reveal a mitochondrial dysfunction in P301L tau transgenic mice. *The Journal of Biological Chemistry*.

[10] Leuner K., Schütt T., Kurz C. (2012). Mitochondrion-derived reactive oxygen species lead to enhanced amyloid beta formation. *Antioxidants and Redox Signaling*.

[11] Valla J., Schneider L., Niedzielko T. (2006). Impaired platelet mitochondrial activity in Alzheimer's disease and mild cognitive impairment. *Mitochondrion*.

[12] Cardoso S. M., Proença M. T., Santos S., Santana I., Oliveira C. R. (2004). Cytochrome c oxidase is decreased in Alzheimer's disease platelets. *Neurobiology of Aging*.

[13] Mancuso M., Filosto M., Bosetti F. (2003). Decreased platelet cytochrome c oxidase activity is accompanied by increased blood lactate concentration during exercise in patients with Alzheimer disease. *Experimental Neurology*.

[14] Shi C., Guo K., Yew D. T. (2008). Effects of ageing and Alzheimer's disease on mitochondrial function of human platelets. *Experimental Gerontology*.

[15] Molina J. A., de Bustos F., Jiménez-Jiménez F. J. (1997). Respiratory chain enzyme activities in isolated mitochondria of lymphocytes from patients with Alzheimer's disease. *Neurology*.

[16] Casademont J., Miró O., Rodriguez-Santiago B., Viedma P., Blesa R., Cardellach F. (2003). Cholinesterase inhibitor rivastigmine enhance the mitochondrial electron transport chain in lymphocytes of patients with Alzheimer's disease. *Journal of the Neurological Sciences*.

[17] Feldhaus P., Fraga D. B., Ghedim F. V. (2011). Evaluation of respiratory chain activity in lymphocytes of patients with Alzheimer disease. *Metabolic Brain Disease*.

[18] Kelly D. P., Scarpulla R. C. (2004). Transcriptional regulatory circuits controlling mitochondrial biogenesis and function. *Genes and Development*.

[19] Scarpulla R. C. (2008). Transcriptional paradigms in mammalian mitochondrial biogenesis and function. *Physiological Reviews*.

[20] Wu Z., Puigserver P., Andersson U. (1999). Mechanisms controlling mitochondrial biogenesis and respiration through the thermogenic coactivator PGC-1. *Cell*.

[21] Cadonic C., Sabbir M. G., Albensi B. C. (2015). Mechanisms of mitochondrial dysfunction in Alzheimer’s disease. *Molecular Neurobiology*.

[22] Ulmer A. J., Scholz W., Ernst M., Brandt E., Flad H. D. (1984). Isolation and subfractionation of human peripheral blood mononuclear cells (PBMC) by density gradient centrifugation on Percoll. *Immunobiology*.

[23] Lanni C., Racchi M., Stanga S. (2010). Unfolded p53 in blood as a predictive signature signature of the transition from mild cognitive impairment to Alzheimer's disease. *Journal of Alzheimer's Disease*.

[24] Lanni C., Racchi M., Mazzini G. (2008). Conformationally altered p53: a novel Alzheimer's disease marker?. *Molecular Psychiatry*.

[25] Acín-Pérez R., Bayona-Bafaluy M. P., Fernández-Silva P. (2004). Respiratory complex III is required to maintain complex I in mammalian mitochondria. *Molecular Cell*.

[26] Bobba A., Amadoro G., Valenti D., Corsetti V., Lassandro R., Atlante A. (2013). Mitochondrial respiratory chain Complexes I and IV are impaired by *β*-amyloid via direct interaction and through Complex I-dependent ROS production, respectively. *Mitochondrion*.

[27] Indo H. P., Yen H.-C., Nakanishi I. (2015). A mitochondrial superoxide theory for oxidative stress diseases and aging. *Journal of Clinical Biochemistry and Nutrition*.

[28] Lezza A. M. S. (2012). Mitochondrial transcription factor A (TFAM): one actor for different roles. *Frontiers in Biology*.

[29] Alvarez-Guardia D., Palomer X., Coll T. (2010). The p65 subunit of NF-kappaB binds to PGC-1alpha, linking inflammation and metabolic disturbances in cardiac cells. *Cardiovascular Research*.

[30] Cherry A. D., Piantadosi C. A. (2015). Regulation of mitochondrial biogenesis and its intersection with inflammatory responses. *Antioxidants & Redox Signaling*.

[31] Gleyzer N., Scarpulla R. C. (2011). PGC-1-related Coactivator (PRC), a sensor of metabolic stress, orchestrates a redox-sensitive program of inflammatory gene expression. *The Journal of Biological Chemistry*.

[32] Piantadosi C. A., Suliman H. B. (2012). Transcriptional control of mitochondrial biogenesis and its interface with inflammatory processes. *Biochimica et Biophysica Acta*.

[33] Schilling J., Lai L., Sambandam N., Dey C. E., Leone T. C., Kelly D. P. (2011). Toll-like receptor-mediated inflammatory signaling reprograms cardiac energy metabolism by repressing peroxisome proliferator-activated receptor *γ* coactivator-1 signaling. *Circulation: Heart Failure*.

[34] Handschin C., Kobayashi Y. M., Chin S., Seale P., Campbell K. P., Spiegelman B. M. (2007). PGC-1*α* regulates the neuromuscular junction program and ameliorates Duchenne muscular dystrophy. *Genes and Development*.

[35] Le Pennec S., Mirebeau-Prunier D., Boutet-Bouzamondo N. (2011). Nitric oxide and calcium participate in the fine regulation of mitochondrial biogenesis in follicular thyroid carcinoma cells. *The Journal of Biological Chemistry*.

[36] Wright D. C. (2007). Mechanisms of calcium-induced mitochondrial biogenesis and GLUT4 synthesis. *Applied Physiology, Nutrition and Metabolism*.

[37] Wright D. C., Geiger P. C., Han D.-H., Jones T. E., Holloszy J. O. (2007). Calcium induces increases in peroxisome proliferator-activated receptor *γ* coactivator-1*α* and mitochondrial biogenesis by a pathway leading to p38 mitogen-activated protein kinase activation. *Journal of Biological Chemistry*.

[38] Acin-Perez R., Salazar E., Brosel S., Yang H., Schon E. A., Manfredi G. (2009). Modulation of mitochondrial protein phosphorylation by soluble adenylyl cyclase ameliorates cytochrome oxidase defects. *EMBO Molecular Medicine*.

[39] Kim S. H., Lu H. F., Alano C. C. (2011). Neuronal Sirt3 protects against excitotoxic injury in mouse cortical neuron culture. *PLoS ONE*.

[40] Wenz T. (2013). Regulation of mitochondrial biogenesis and PGC-1*α* under cellular stress. *Mitochondrion*.

[41] Strazielle C., Jazi R., Verdier Y., Qian S., Lalonde R. (2009). Regional brain metabolism with cytochrome c oxidase histochemistry in a PS1/A246E mouse model of autosomal dominant Alzheimer's disease: correlations with behavior and oxidative stress. *Neurochemistry International*.

[42] Moreira P. I., Santos M. S., Oliveira C. R. (2007). Alzheimer's disease: a lesson from mitochondrial dysfunction. *Antioxidants and Redox Signaling*.

[43] Sompol P., Ittarat W., Tangpong J. (2008). A neuronal model of Alzheimer's disease: an insight into the mechanisms of oxidative stress-mediated mitochondrial injury. *Neuroscience*.

[44] Buizza L., Cenini G., Lanni C. (2012). Conformational altered p53 as an early marker of oxidative stress in Alzheimer's disease. *PLoS ONE*.

[45] Michel S., Wanet A., De Pauw A., Rommelaere G., Arnould T., Renard P. (2012). Crosstalk between mitochondrial (dys) function and mitochondrial abundance. *Journal of Cellular Physiology*.

[46] Rodriguez-Enriquez S., Kai Y., Maldonado E., Currin R. T., Lemasters J. J. (2009). Roles of mitophagy and the mitochondrial permeability transition in remodeling of cultured rat hepatocytes. *Autophagy*.

[47] Malik A. N., Czajka A. (2013). Is mitochondrial DNA content a potential biomarker of mitochondrial dysfunction?. *Mitochondrion*.

[48] Larsson N.-G., Wang J., Wilhelmsson H. (1998). Mitochondrial transcription factor A is necessary for mtDNA maintenance and embryogenesis in mice. *Nature Genetics*.

[49] Ekstrand M. I., Falkenberg M., Rantanen A. (2004). Mitochondrial transcription factor A regulates mtDNA copy number in mammals. *Human Molecular Genetics*.

[50] Gong B., Chen F., Pan Y. (2010). SCFFbx2-E3-ligase-mediated degradation of BACE1 attenuates Alzheimer's disease amyloidosis and improves synaptic function. *Aging Cell*.

[51] Qin W., Haroutunian V., Katsel P. (2009). PGC-1*α* expression decreases in the Alzheimer disease brain as a function of dementia. *Archives of Neurology*.

[52] Sheng B., Wang X., Su B. (2012). Impaired mitochondrial biogenesis contributes to mitochondrial dysfunction in Alzheimer's disease. *Journal of Neurochemistry*.

[53] Zhang Q., Yu J.-T., Wang P. (2011). Mitochondrial transcription factor A (TFAM) polymorphisms and risk of late-onset Alzheimer's disease in Han Chinese. *Brain Research*.

[54] Alvarez V., Corao A. I., Alonso-Montes C. (2008). Mitochondrial transcription factor a (TFAM) gene variation and risk of late-onset Alzheimer's disease. *Journal of Alzheimer's Disease*.

[55] Chaturvedi R. K., Beal M. F. (2013). Mitochondrial diseases of the brain. *Free Radical Biology and Medicine*.

[56] Wallace D. C. (2005). A mitochondrial paradigm of metabolic and degenerative diseases, aging, and cancer: a dawn for evolutionary medicine. *Annual Review of Genetics*.

